# Supplementary material for: Predicting Visual Acuity Deterioration and Radiation-Induced Toxicities after Brachytherapy for Choroidal Melanomas
Source: Cancers (Basel). 2019 Aug 6;11(8):1124. doi: 10.3390/cancers11081124 (PMC6721463; doi:10.3390/cancers11081124)
Supplement: Supplementary file 1 [file cancers-11-01124-s001.pdf]

SUPPLEMENTARY MATERIAL

Analysis plan

Complication/outcome	Dose to structure*	Clinical factors	Analysis	Competing risks
**Visual acuity (VA) deterioration**<br>i. Pre-treatment VA ≤ 0.5 logMAR. Post-treatment VA increase of 0.3 logMAR compared to initial<br>ii. All patients regardless of initial visual acuity. Negative post-treatment VA change of 0.3 logMAR compared to pre-treatment<br>iii. The first negative change after 3 months in post-treatment VA of 0.3 logMAR compared to pre-treatment	Retina (surface)<br>Macula (surface)<br>Optic disc (surface)<br>Globe (volume)<br>Lens (volume)	Age, gender, tumour height, tumour-optic disc distance, follow-up time (for logistic regression), treatment time	Analysis i. and ii. were performed from Cox regression<br><br>Analysis iii. was performed by logistic regression	Censoring: death from any cause, lost to follow-up, relapse or enucleation, tumour under the macula in follow-up, detached macula in follow-up, tumour covering the optic disc in follow-up
**Maculopathy**	Retina (surface)<br>Macula (surface)<br>Optic disc (surface)<br>Globe (volume)<br>Lens (volume)	Age, gender, tumour height, tumour-optic disc distance, treatment time, tumour under the macula at baseline, detached macula at baseline	Cox regression analysis	Censoring: death from any cause, lost to follow-up, relapse or enucleation, tumour under the macula in follow-up, detached macula in follow-up
**Optic neuropathy**	Retina (surface)<br>Macula (surface)<br>Optic disc (surface)<br>Globe (volume)	Age, gender, tumour height, tumour-optic disc distance, treatment time,	Cox regression analysis	Censoring: death from any cause, lost to follow-up, relapse or

		tumour covering the optic disc at baseline		enucleation, tumour covering the optic disc in follow-up
**Retinal detachment (post-treatment)**	Retina (surface)	Age, gender, tumour height, tumour-optic disc distance, treatment time	Cox regression analysis	Censoring: death from any cause, lost to follow-up, relapse or enucleation
**Ocular hypertension**	Retina (surface)<br>Optic disc (surface)	Age, gender, tumour height, tumour-optic disc distance, treatment time	Cox regression analysis	Censoring: death from any cause, lost to follow-up, relapse or enucleation
**Vascular obliteration**	Retina (surface)<br>Macula (surface)<br>Optic disc (surface)<br>Globe (volume)	Age, gender, tumour height, tumour-optic disc distance, treatment time	Cox regression analysis	Censoring: death from any cause, lost to follow-up, relapse or enucleation
**Cataract**	Retina (surface)<br>Lens (volume)	Age, gender, tumour height, tumour-optic disc distance, treatment time	Cox regression analysis	Censoring: death from any cause, lost to follow-up, relapse or enucleation

Table S1: Analysis plan. For each late complication we pre-specified variables to include in the Lasso selection process including dose to specific structures and clinical characteristics. Furthermore, we defined analysis methods and competing events for each of the late complications. *Dose to specific areas/volumes of the structure ($D_{2\,\%}$, $D_{20\,\%}$, $D_{50\,\%}$, $D_{98\,\%}$) was used in the model. Furthermore, we included area/volume that received a specific dose (A/V_{200Gy}, A/V_{100Gy}, A/V_{80Gy}, A/V_{50Gy}, A/V_{20Gy}, A/V_{10Gy}).

Visual acuity supplementary data

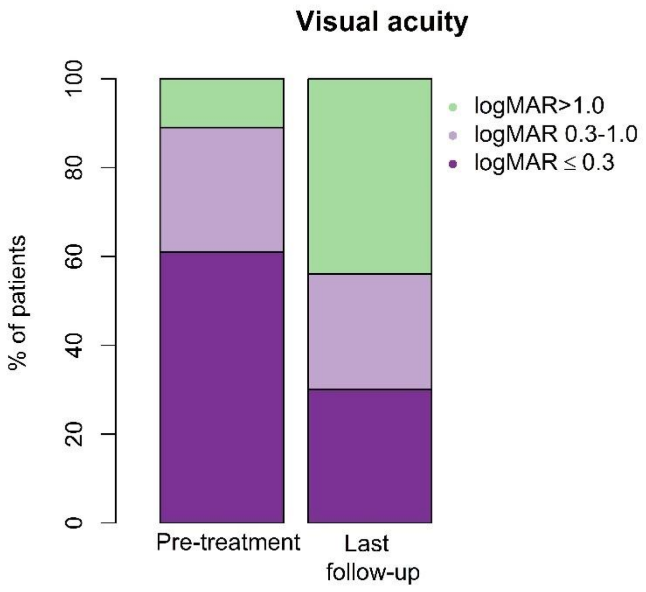

Figure S1: Bar chart of pre-treatment visual acuity and last visual acuity measure for all patients.

The overall risk of visual acuity loss is illustrated in the Kaplan-Meier curves in Figure S2 for both visual acuity deterioration (group 1) and pre-treatment visual acuity loss (group 2).

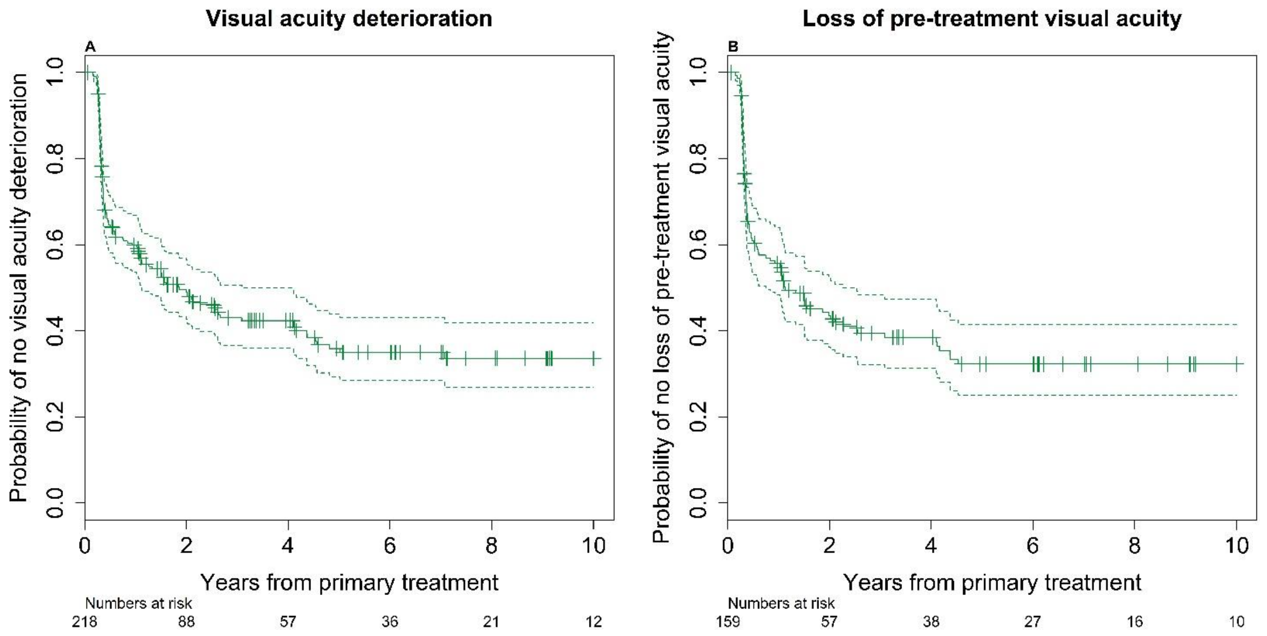

Figure S2: Kaplan-Meier analysis for visual acuity deterioration (group 1) and loss of pre-treatment visual acuity (group 2). Dotted lines illustrate the 95% confidence intervals; the crosses illustrate censored patients.

Logistic regression

For explorative purposes we performed logistic regression analyses for visual acuity loss for both visual acuity deterioration (group 1) and pre-treatment visual acuity loss (group 2). In both analyses, visual acuity was defined as a negative difference of ≥0.3 logMAR between the pre-treatment measure and the measure at the last regular assessment.

The odds ratios are listed in Table B1.

	**Odds ratio (95 % CI)**
**Visual acuity deterioration**	
Optic disc-tumour distance	0.88 (0.78-0.99)
Macula A_{10Gy} *	1.10 (0.96-1.27)
Macula A_{50Gy} *	0.93 (0.60-1.47)
Macula A_{80Gy} *	1.17 (0.73-1.87)
**Loss of pre-treatment visual acuity**	No variables selected

Table S2: Odds ratios from logistic regression analyses for visual acuity deterioration and loss of pre-treatment visual acuity.

The dose-response model for the logistic regression analysis is illustrated in Figure S3A and in Figure S3B for three specific optic disc-tumour distances.

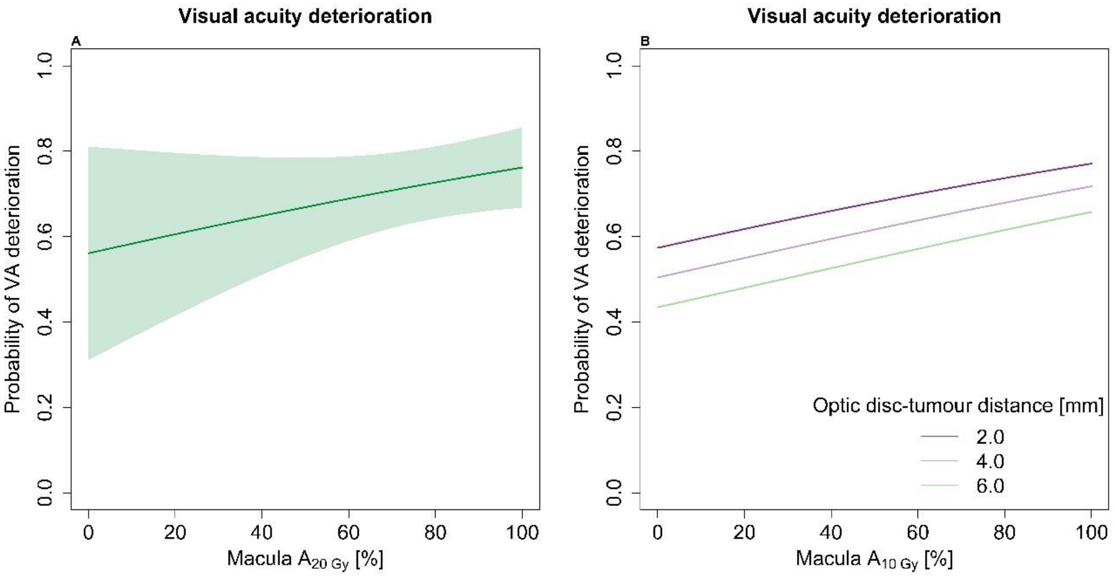

Figure S3: A) Dose response of visual acuity deterioration as a function of macula A_{10Gy}. The model adjusts for optic disc-tumour distance (2.4 mm), macula A_{50Gy} (26 %) and macula A_{80Gy} (8 %). The shaded area indicates the 95 % confidence intervals. B) Dose response of visual

acuity deterioration as a function of macula V_{10Gy} for three specific optic disc-tumour distances (2, 4 and 6 mm).

Model performance for the logistic regression analysis was assess using Hosmer-Lemeshow. The results showed acceptable calibration.

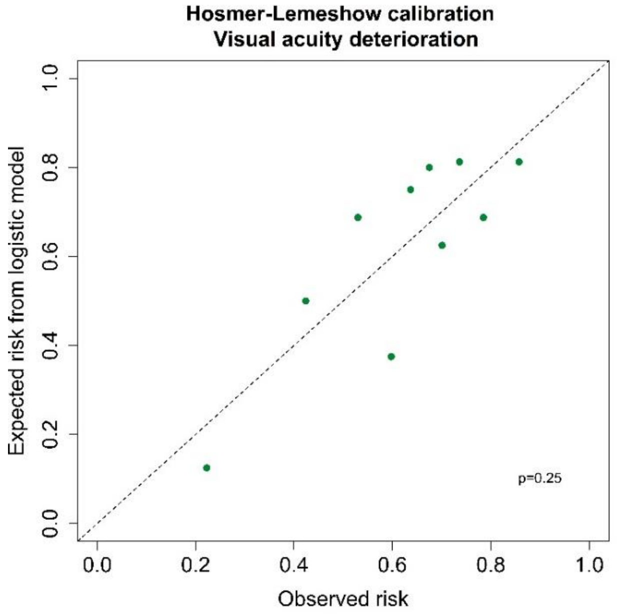

Figure S4: Hosmer-Lemeshow calibration curve

Late complications supplementary

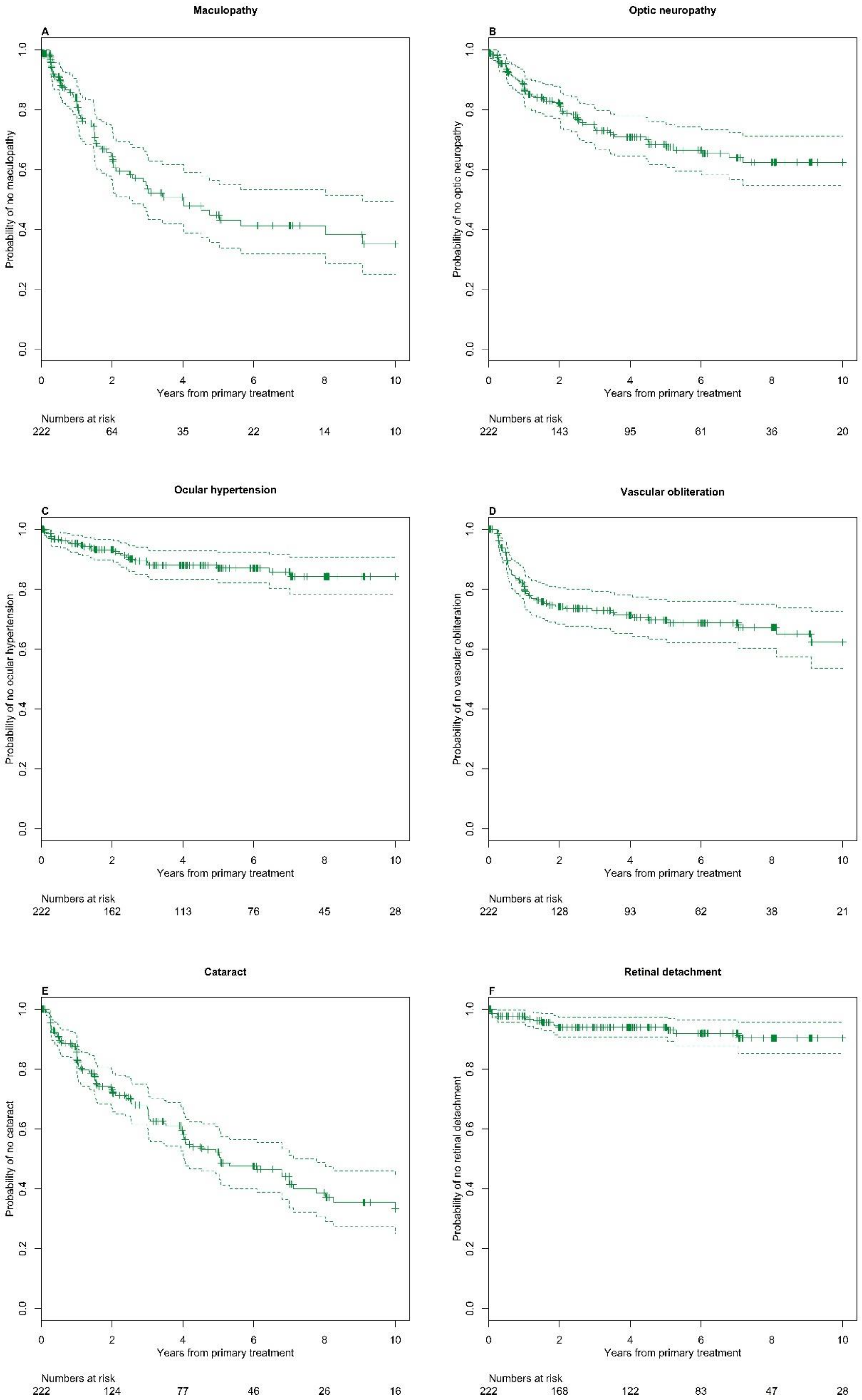

Maculopathy
A
Probability of no maculopathy
Years from primary treatment
Numbers at risk
222 64 35 22 14 10
Optic neuropathy
B
Probability of no optic neuropathy
Years from primary treatment
Numbers at risk
222 143 95 61 36 20
Ocular hypertension
C
Probability of no ocular hypertension
Years from primary treatment
Numbers at risk
222 162 113 76 45 28
Vascular obliteration
D
Probability of no vascular obliteration
Years from primary treatment
Numbers at risk
222 128 93 62 38 21
Cataract
E
Probability of no cataract
Years from primary treatment
Numbers at risk
222 124 77 46 26 16
Retinal detachment
F
Probability of no retinal detachment
Years from primary treatment
Numbers at risk
222 168 122 83 47 28

Figure S5: Kaplan-Meier for late complications. A) maculopathy, B) optic neuropathy, C) ocular hypertension, D) Vascular obliteration, E) cataract and F) retinal detachment. Dotted lines illustrate the 95% confidence intervals; the crosses illustrate censored patients.

Model performance

Complication	5-year c-index	5-year Brier score
Visual acuity deterioration	65.5 %	0.187
Maculopathy	65.8 %	0.182
Optic neuropathy	78.7 %	0.149
Vasculopathy	69.0 %	0.176
Cataract	64.7 %	0.218

Table S3: 5-year concordance indices and Brier scores for each late complication

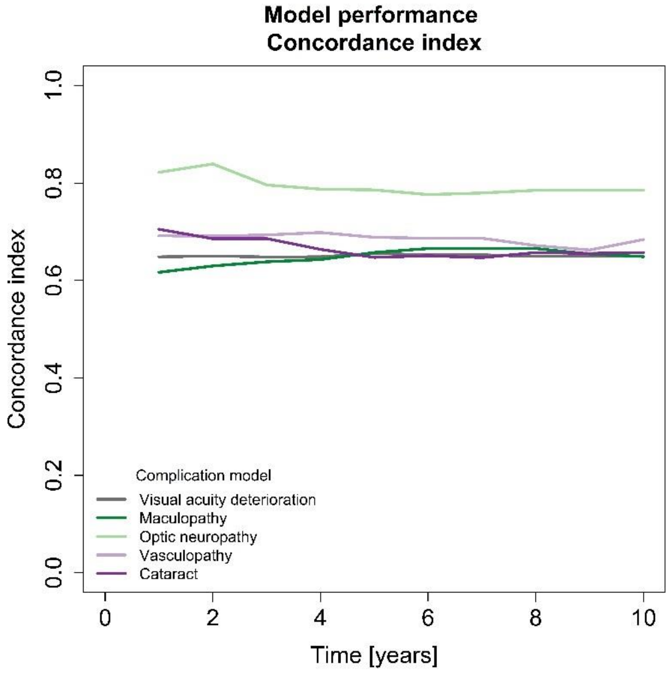

Figure S6: Concordance indices

Median dose area/volume histograms

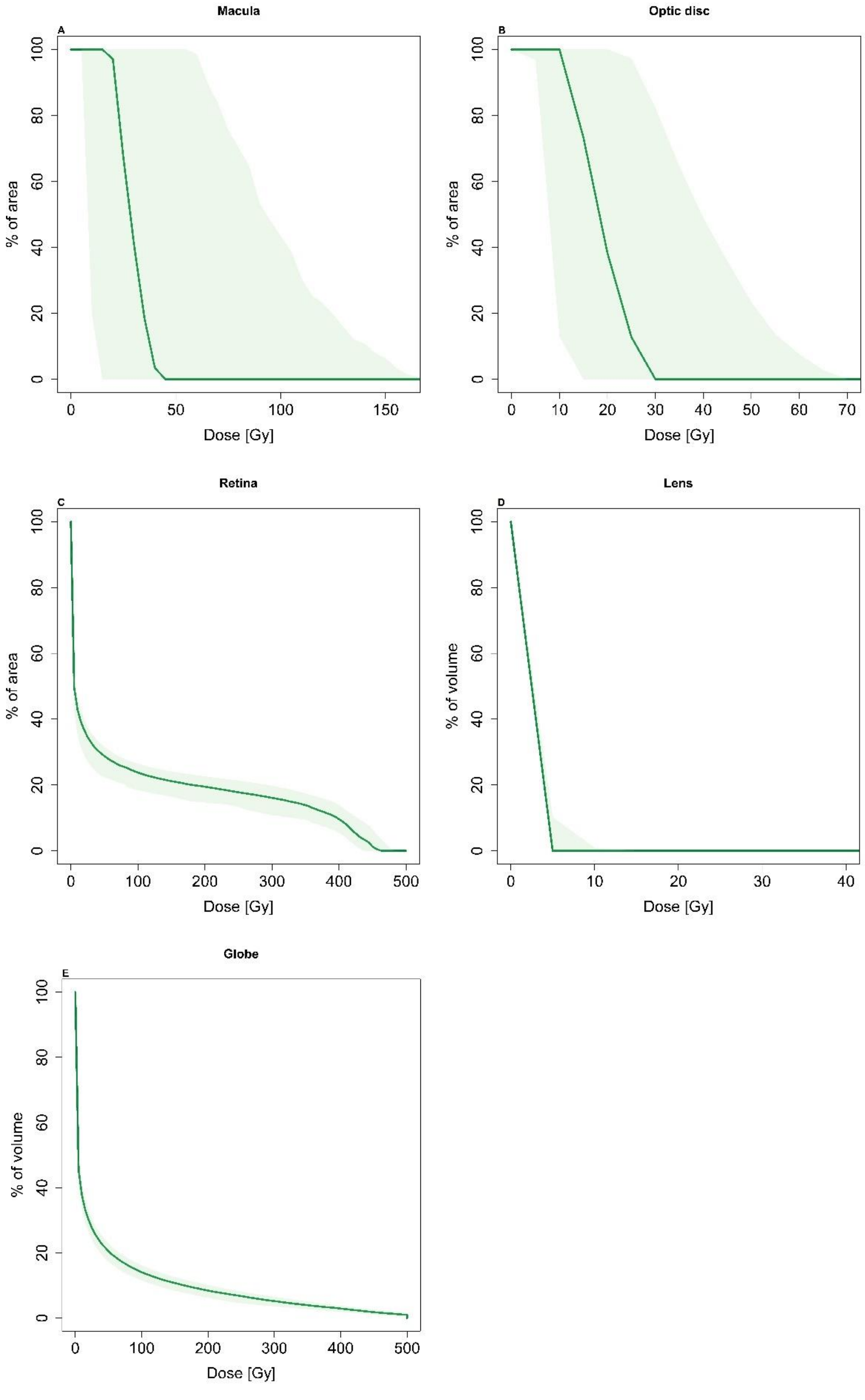

Figure S7: Median dose area histograms for A) macula, B) optic disc, C) Retina. Median dose volume histograms for D) Lens and E) globe. The shaded light green area illustrates the 25 % and 75 % quartiles, respectively.
